# Symptom recognition and health care seeking among immigrants and native Swedish patients with heart failure

**DOI:** 10.1186/1472-6955-7-9

**Published:** 2008-06-30

**Authors:** Azar Hedemalm, Maria Schaufelberger, Inger Ekman

**Affiliations:** 1 Institute of Health and Care Sciences, The Sahlgrenska Academy at Göteborg University, Göteborg, Sweden; 2 Departments of Emergency and Cardiovascular Medicine, Sahlgrenska Academy at Göteborg University, Göteborg, Sweden

## Abstract

**Background:**

It is not known what patient perceptions or beliefs lead to beneficial decisions or response patterns in symptom interpretation among heart failure (HF) patients, especially immigrants. The aim of this study was to explore and compare symptom recognition and health care seeking patterns among immigrants and native Swedes with HF.

**Methods:**

The study used a qualitative design. Semi-structured interviews were conducted with 42 patients with HF, of whom 21 were consecutively selected immigrants and 21 were randomly selected Swedish patients. The interviews were analysed using content analysis.

**Results:**

A majority of the immigrant patients sought health care for symptoms and signs, such as breathing difficulties, fatigue and swelling. Twice as many immigrants as Swedes were unaware of "what the illness experience entailed" and which symptoms indicated worsening of HF.

**Conclusion:**

The symptoms that patients sought care for, were similar among immigrants and Swedes. However, when interpreting symptoms more immigrants were unaware of the connection between the symptoms/signs and their HF condition. More tailored educational interventions might improve recognition of worsening symptoms in immigrant patients with chronic heart failure.

## Background

Heart failure (HF) is a common condition contributing to significant morbidity and mortality in Western societies. It significantly impacts all aspects of quality of life, particularly impairing patients' mobility and performance of daily activities [1]. Cultural differences exist among patients with HF in the interpretation of symptoms, hospital readmission rates, and functional status [2]. The meaning that patients assign to symptoms and illness can have profound implications for their wellbeing. This meaning is influenced by culture, gender, experience, learning and beliefs, and all of these influence the individual's assessment of symptoms [3]. Family systems, coping styles, health beliefs, behaviours and practices influence the individual response to chronic illness, often as a consequence of a chronic disease. Illness represents culturally shaped reactions to a certain discomfort which mirrors the way an individual perceives, experiences and copes with it [4]. According to Helman, illness can be perceived as caused by external or internal factors, e.g., factors related to the individual, social relations, natural or supernatural forces [5]. While illness reflects a personal and social experience of health status represented as symptoms, disease is the physiological disturbance with signs as its clinical hallmarks [6]. Cultural differences exist also in explanations for the cause of disease and interpretation of symptoms. Such differences may lead to different responses to the illness, e.g., different self care measures or health care seeking behaviours.

Symptoms are sensations that signal a disturbance in normal body function to the individual. If these sensations cannot be recognised and management strategies are lacking, the individual's response may be inadequate or insufficient. Recognising the symptoms as a representation of unfamiliar sensations and responding adequately are behaviours which are related to the individual's cognitive, communicative or physical capabilities [3]. Previous studies on symptoms in HF have reported up to 45 different symptoms [7,8]. In a recent study, the majority of the patients with HF (58 %) sought emergency care due to symptoms and 70% of those who delayed seeking care for exacerbation of HF explained that they chose to "wait and see". This strategy may imply a failure to recognise symptoms of deterioration [9] as medical attention is often sought only when acute or life-threatening symptoms occur [10,11].

Appropriate health care seeking decisions are based on symptom recognition. Interpretation and evaluation of the experience requires sufficient knowledge about the disease, its treatment and recommended self care. This knowledge may also enable patients to develop their own management strategies. Several researchers have stated that the assessment and interpretation of sensations are influenced by gender, culture, beliefs, experience and learning [12-14].

A review of the literature shows an under-representation of women, elderly, ethnic minorities and immigrants in clinical trials. The inclusion of these groups is imperative for determining appropriate treatments for these groups. However, studies involving HF patients have generally not focused on patient beliefs, perspectives and experiences of care and treatment, but rather on the pathophysiology of the condition, clinical factors or risk factors, epidemiology, readmissions and economical costs.

Immigrants in this study are defined as those who are born abroad, have another mother tongue than Swedish and are living in Sweden at present. This definition is in contrast to several other research designs which are built up on racial differences and classifies the participants as for example Asian, Black, White and Caucasian [15]. This kind of classification is not distinct enough because people classified as belonging to a racial or ethnical group might not necessary be immigrants and they may well have be living in the county for many generations. The word race is not an adequate variable in study of differences between human populations since the lion's share of human gene variation falls within, not between populations. Therefore the term "race" remains a social construct with social meaning and influence on the health without having any biological or genetic basis [16].

Due to an extensive global migration, Sweden has become a multicultural society with more than 120 different nationalities represented in the country. It is not known what patient perceptions or beliefs lead to beneficial decisions or response patterns in symptom interpretation among HF patients, especially immigrants. Moreover, knowledge about health care seeking patterns in immigrants is scarce.

## Objective

The aim of this study was to explore and compare symptom recognition and health care seeking patterns among immigrants and native Swedes with HF.

## Method

### Design and setting

This study was explorative and comparative, and used a qualitative design with semi-structured interviews. The setting was a major Swedish University Hospital serving a large urban, multiethnic community. Approval to conduct this study was obtained from the Regional Ethical Review Board, Göteborg University. Participants gave their written informed consent to participate before the inclusion and they were assured confidentiality.

### Patients and methods

This study is a part of bigger research project conducted between April 2004 and May 2006 at a university hospital in Western Sweden, serving several multicultural neighbourhoods. The patient sample comprised 42 participants who were included in the study at the visit to emergency department (ED), inpatient cardiac and HF clinic at the hospital admission or 72 hours after the admission. Twenty one were consecutively selected immigrants and a comparison group of 21 Swedish patients was included also consecutively among 128 Swedes from the inclusion list for the study that was registered by date. Every fifth patient was included from this list in the comparison group, if they met inclusion and exclusion criteria. The inclusion criterion was either hospital admission for acute HF or worsening of chronic heart failure (CHF) at the time of the study. Exclusion criteria were communication difficulties (primarily due to stroke, dementia, hearing difficulties, confusion or disorientation), alcohol abuse, participation in other ongoing studies and more or less permanent institutionalisation.

Data were obtained from semi-structured patient interviews were performed by trained interviewers. The interviews which lasted 15–30 minutes were performed either at the ED or the ward by handwriting of pragmatic reasons. Immigrants were defined as persons born abroad with a first language other than Swedish. The diagnosis of HF was verified by one of the authors, a senior cardiologist, from patient records based on the European Society of Cardiology guidelines for the definition and diagnosis of HF [17]. Interviews with the immigrants were performed with the help of five professional authorized interpreters. The interview guide addressed health seeking patterns and probable delays. We defined delay as having symptoms due to CHF but not seeking care until at least one symptom is unbearable.

The interview questions were:

a) Why did you seek care?

b) What symptoms did you experience?

c) Did you know what kind of illness it was?

d) Do you think you should have sought health care earlier?

Patients were asked about demographic factors (age, sex, country of origin) and behavioural risk factors (smoking, overweight and alcohol use). Patients' medical history (e.g. history of heart failure, co-morbidities, medications and ejection fraction, EF) was obtained from their patient records. Body weight was measured on admission and the New York Heart Association (NYHA) classification was assessed by researchers and assisting research nurses.

### Data analysis

The interviews were analysed using content analysis and subcategories were derived inductively from the text material [18]. In this qualitative content analysis, all answers from each patient group were merged into a single text, which was then read and re-read to identify units of analysis related to the research question. In the next step, the units of analysis were lifted out from the text and condensed to subcategories and then coded into categories (Table 1). In the final step these categories were compared between the groups regarding how often they occurred. Derived categories are presented as frequencies. To enhance the credibility of the analysis, the text was independently analysed by two of the authors (AH and IE). There were few disagreements, which were discussed to reach consensus about categories. Descriptive statistics has been performed for demographic and clinical data. Quantitative continuous data were tested using the non-parametric Mann-Whitney U-test and category differences were evaluated using chi-square tests.

**Table 1:** Illustration of data analysis

**Unit of analysis**	**Subcategory**	**Category**
The legs felt bloated, like balloons, water in the legs, Bowel swelling, I felt that my face and body were swollen	*Extremity swelling/General swelling*	Swelling
Not slept all night and was sitting in bed the whole day, I could not lie down and sleep because of breathing difficulties	*Breathing difficulties at night*	Orthopnea
Difficult to breathe, Breathing was difficult and almost suffocating	*Abnormal breathing*	Breathing difficulties
I knew that it was the heart. suspected heart infarction	*It was the heart*	The heart
My wife and family noticed the worsening, Home care personnel recommended me to seek acute care	*Not recognise the symptom*	Symptom recognition

## Results

### Participants

The immigrant group originated from 12 countries. Eighteen patients (86 %) were from European countries and three (14 %) from non-European countries. Nine patients (43 %) needed an interpreter to communicate with the health care professionals. The number of women in the immigrant and Swedish groups was ten and eight respectively. No significant differences were found between the immigrant and Swedish groups in severity of heart failure, as measured by EF, or functional status, assessed by NYHA classification. Haemoglobin level, baseline weight or HF medications did not differ significantly between the groups. In both groups, 75% had a compulsory school level of education and a mean of two co-morbidities. No significant differences were found regarding co-morbidities (hypertension, diabetes, lung disease and atrial fibrillation: table 2) or in physical function (impaired vision, hearing or need of walking assistance). Eleven patients in each group had previously had a follow-up visit at the nurse-led HF clinic or other follow ups due to HF. Three of these patients needed interpreters but just one had a professional interpreter noted in the patient records.

**Table 2:** Patient characteristics

	**Immigrants ** (N = 21)	**Swedes ** (N = 21)
**Demographics**		
Age, mean (± SD)	79 (5)	70 (8)
Gender, female (n)	10	8
Living alone	9	15
**Education level **(n)		
0–9 yrs	15	16
< 9 yrs	1	2
< 12 yrs	1	2
Missing data	4	1
**Risk factors **(n)		
Smoking	0	3
Never smoked	9	7
Ex-smokers	12	11
Alcohol consumption	8	13
**Clinical data**, mean (± SD)		
NYHA-class	3 (0.4)	3 (0.4)
EF %	31 (7)	36 (12)
Haemoglobin	135 (12)	132 (13)
**Comorbidities on admission**, (n)		
Ischemic heart disease	12	15
Artrial fibrillation	12	10
Valvular heart disease	8	7
Hypertension	9	12
Lung disease	3	4
Diabetes	5	9
**Medication on admission**, (n)		
ACE-inhibitors	12	9
ARB	2	7
Beta blockers	14	19
Diuretics	17	19
Anticoagulants	12	16

EF; ejection fraction, ACE; Angiotensin converting enzyme

ARB; angiotensin receptor blocker

### Interviews

#### Why did you seek health care and what symptoms did you experience?

The majority of both patient groups (immigrants 81 %; Swedes 67 %) sought medical attention due to symptoms, such as breathing difficulties, orthopnea, sleeping disorders, fatigue and swelling (legs or body), feelings of fear and anxiety, dizziness, pain, lack of appetite, cough and palpitation (table 3). All patients reported more than one symptom. Those who had not recognised worsening symptoms or did not experience them as discomforting were encouraged by their family members or health care professionals to seek care (immigrants 19 %; Swedes33%).

**Table 3:** Reasons for seeking health care

**What did you seek for? **(n)	**Immigrants ** (N = 21)	**Swedes ** (N = 21)
Breathing difficulties	19	19
Swelling, (legs or other parts of the body)	13	14
Fatigue	11	8
Orthopnea	7	9
Sleeping difficulties due to orthopnea	7	9
Pain, (chest or other parts of the body)	9	6
Fear and anxiety	4	5
Reduced physical capacity	4	7
Feeling unwell	5	1
Balance difficulties	3	5
Palpitation	3	2
Cough	2	1
Constipation	1	-
Lack of appetite	-	1
**Did you know what it was about? **(n)		
Yes	11	15
No	10	5
Uncertain	2	2
**It was about.... **(n)		
"The heart"	7	10
Medications	2	-
Water retention	2	2
Oxygen shortage	1	-

#### Did you know what the underlying cause of the symptoms was?

Eleven of the immigrants and fifteen of the Swedes stated that they had recognised the symptoms they sought care for, but some of the patients still expressed some uncertainty in their answers. Some of these patients also stated e.g. that "the cause may be my diabetes or heart", "the smoking", "angina", "could be the heart". Twice as many immigrants (n = 10) as Swedes (n = 5) stated that they had no idea of what the underlying cause of their illness was. Statements explaining reasons for patients seeking care are listed in table 3.

#### Do you think you should have sought health care earlier?

An equal number (n = 12; 57 %) of immigrants and Swedes were aware of their delay in seeking care from the onset of the symptoms to arrival at hospital. Half of the patients (9 immigrants and 12 Swedes) gave explanations for not having sought care earlier (table 4). Two of the Swedish patients did not experience the condition as discomforting or disconcerting.

**Table 4:** Do you think you should have sought health care earlier?

**Statements**	**Immigrants ** (N = 21)	**Swedes ** (N = 21)
**Do you think you should have ** **sought health care earlier? **(n)		
Yes	12	12
No	9	8
Missing data	-	1
**Reasons for not seeking care earlier **(n)		
Waited for follow-up visit	1	1
Used telephone counselling	-	2
Hoped for improvements	2	1
Long waiting time at ED	1	-
Seeking care puts too much strain on me	1	-
Expense	1	-
Had nobody to accompany me	1	-
Other reasons	-	1
No comment	12	9

**ED; **Emergency department

## Discussion

Consistent with previous research [9], we found that symptoms were the main reason for patients to seek health care and that there was uncertainty in how to interpret the symptoms and its relation to HF, especially among immigrants. Prior research findings have also confirmed uncertainty about the condition and its management among patients with HF [19]. Patients with HF are often elderly with multiple chronic diseases, which may complicate symptom interpretation further. Symptom recognition in relation to different diseases may be even more aggravated in immigrants since their accessibility to sources of information about HF may be limited. Immigrant patients in this study were older than Swedes, which may indicate undiagnosed or documented cognitive impairments. Previous studies have confirmed a relationship between HF and cognitive impairment [20]. Such impairments could be a reason for misinterpretation of symptoms related to HF condition.

Underuse of interpreters and fewer follow-ups among immigrants after the first visit at the nurse-led HF clinic might explain their poor knowledge about the condition [21]. Other possible explanations are that communication between hospital health providers and those in primary care may be inadequate and possible shortcomings in handover and follow-up routines at discharge may lead to discontinuity in the care process. Assistive supportive systems in HF management should be continued after discharge and be linked to nurse-led outpatient HF clinics or primary care clinics to facilitate easy access to health care professionals. In order to reduce delays in seeking care, primary care HF clinics with fast access to health care and interpreters, e.g. by phone or internet could be one option. Such clinics would facilitate for immigrants with language deficiencies to seek immediate counselling and care without having to first overcome the barrier of booking an appointment by telephone.

Despite presence of worsening symptoms patients in both groups delayed seeking health care, which is in consistency with previous studies [22]. One reason for patient delays could be that the patients simply failed to identify early symptoms of worsening HF. Other factors contributing to intentional or non-intentional delays in health care seeking should be thoroughly explored among both patient groups. In this regard, characteristics such as the individual's world view, the causal explanation of the disease from a patient perspective, the meaning of illness and cultural metaphors assigned to the illness and disease should also be considered [23]. However, patients' perceptions of their role and ability to influence the condition and perform self-care are also important. Other patient-related factors, such as lack of knowledge about how the health care system works (how to enter and use it), can also explain delays in seeking health care. Moreover, lack of language skills or dependence on others to get to hospital or primary care clinics may contribute to delays [24]. Our patients may also have sought other informal care prior to hospital admissions [25].

Previous findings at a HF clinic showed that biological and medical aspects of care were more often recorded than psychosocial aspects [21], which may confirm that patient perspectives have lower priority, or possibly that our existing documentation system is focused on bio-physical aspects. Health care professionals often give higher priority to assessing signs and addressing disease management than to assessing illness experience and its meaning and consequences on patients' daily lives [17,23]. In prioritising these aspects of care, the health care professionals may inadvertently ignore the individual patient's needs, resources and priorities. According to recommendations from the European Society of Cardiology for HF discharge planning, a multi-disciplinary team approach should be used to discuss with patients what early signs and symptoms indicate worsening of the condition. The team can also provide intensive education and counselling, conduct inpatient and outpatient follow-ups, and optimise medical therapy [17]. However, it appears that the last goal, i.e. optimising medical therapy, is the primary one at all care levels in HF management.

As in most other incurable and chronic conditions, an important treatment goal for HF is to improve the patient's quality of life. Consequently, preventive and health-promoting interventions should also be consigned higher priority in order to address informational needs and to assist patients in developing their own self management strategies based on their own beliefs and preferences.

Assessment of symptoms is considered to be important for the diagnosis and choice of treatment in HF [17]. Therefore it is important to assess patient's symptom experience. Some of our immigrant patients in the present study described their experience of illness as "feeling unwell" and gave this as one of the reasons for seeking acute care. This description may reflect language deficiencies or difficulties to find the right words and expressions among immigrants. Because it is a diffuse description, its significance is not easily interpreted by health care professionals. Nor is it precise enough to permit the assessment of its relevance to the management of the condition. Health care professionals need not only elicit the help of professional interpreters, but also thoroughly explore unclear descriptions of patients' illness experiences during the history taking session. Asking more attendant questions may enable health care professionals to better grasp the patient's experiences of burden and discomfort, especially when language barriers exist. This is imperative in order to gain an understanding of the impact of the illness experience on the patient's daily life so as to be able to provide patient-centred care related to the disease. It may also hinder misunderstandings about the symptoms of HF and thus enhance disease management and symptom relief.

### Limitation of the study

The generalisability of our findings to the immigrant population is limited by the heterogeneity of both our immigrant patient sample and the general immigrant population. This limitation is also the strength of the study since it enabled us to sample a wide variety of perspectives and explanations of different peoples. Due to pragmatic reasons we did not perform external validation of the content analysis, which could be another limitation of the study.

## Conclusion and implications

*Conclusion: *Symptom interpretation was dissimilar between immigrants and Swedes, more immigrants were unaware of the connection between the symptoms and signs and their HF condition. More tailored educational interventions might improve recognition of worsening symptoms in patients with chronic heart failure.

## Competing interests

The authors declare that they have no competing interests.

## Authors' contributions

AH: Study design, data collection, data analysis, preparing initial draft and the final draft of the manuscript. MS: Study design, contributed to the revising the final draft. IE: Study design, data analysis, contributed to the revising the final draft. All authors read and approved the final manuscript.

## Pre-publication history

The pre-publication history for this paper can be accessed here:


